# Comparative proteomic analysis of QTL *CTS-12* derived from wild rice (*Oryza rufipogon* Griff.), in the regulation of cold acclimation and de-acclimation of rice (*Oryza sativa* L.) in response to severe chilling stress

**DOI:** 10.1186/s12870-018-1381-7

**Published:** 2018-08-10

**Authors:** Weijian Cen, Jianbin Liu, Siyuan Lu, Peilong Jia, Kai Yu, Yue Han, Rongbai Li, Jijing Luo

**Affiliations:** 1 State Key Laboratory for Conservation and Utilization of Subtropical Agro-bioresources, Guangxi University, Nanning, 530004 China; 2 College of Life Science and Technology, Guangxi University, Nanning, 530004 China; 3 College of Agriculture, Guangxi University, Nanning, 530004 China; 4 Shanghai MHelix BioTech Co., Ltd, Shanghai, 201900 People’s Republic of China

**Keywords:** Wild rice, Chilling stress, Recovery, Comparative proteomics, Photosynthetic proteins, Ribosomal proteins, Cold acclimation, de-acclimation

## Abstract

**Background:**

Rice (*Oryza sativa* L.) is a thermophilic crop vulnerable to chilling stress. However, common wild rice (*Oryza rufipogon* Griff*.*) in Guangxi (China) has the ability to tolerate chilling stress. To better understand the molecular mechanisms underlying chilling tolerance in wild rice, iTRAQ-based proteomic analysis was performed to examine *CTS*-*12*, a major chilling tolerance QTL derived from common wild rice, mediated chilling and recovery-induced differentially expressed proteins (DEPs) between the chilling-tolerant rice line DC90 and the chilling-sensitive 9311.

**Results:**

Comparative analysis identified 206 and 155 DEPs in 9311 and DC90, respectively, in response to the whole period of chilling and recovery. These DEPs were clustered into 6 functional groups in 9311 and 4 in DC90. The majority were enriched in the ‘structural constituent of ribosome’, ‘protein-chromophore linkage’, and ‘photosynthesis and light harvesting’ categories. Short Time-series Expression Miner (STEM) analysis revealed distinct dynamic responses of both chloroplast photosynthetic and ribosomal proteins between 9311 and DC90.

**Conclusion:**

*CTS-12* might mediate the dynamic response of chloroplast photosynthetic and ribosomal proteins in DC90 under chilling (cold acclimation) and recovery (de-acclimation) and thereby enhancing the chilling stress tolerance of this rice line. The identified DEPs and the involvement of *CTS-12* in mediating the dynamic response of DC90 at the proteomic level illuminate and deepen the understanding of the mechanisms that underlie chilling stress tolerance in wild rice.

**Electronic supplementary material:**

The online version of this article (10.1186/s12870-018-1381-7) contains supplementary material, which is available to authorized users.

## Background

Chilling stress is one of the most common abiotic stresses and can cause severe injury in all stages of rice growth with seedling stage being one of the most vulnerable to chilling stress. The optimal temperature for rice growth is 25–35°C. Low temperature not only causes severe injury to early-season cold-sensitive rice cultivars in the spring but also leads to substantial yield loss of late-season rice in the autumn. With the increase in global climate anomalies, chilling stress occurs frequently, and the range of its influence is widening [1]. Therefore, understanding the mechanisms that underlie chilling tolerance and to breeding chilling-tolerant cultivars for rice production are urgent goals.

Chilling stress triggers a series of changes in physiological and molecular processes [2] and results in the accumulation of reactive oxygen species (ROS) in plant cells. The accumulating ROS cause oxidative stress, which damages the plant cell membrane, decreases enzyme activity, and inhibits the rate of photosynthesis and protein translation [3, 4]. Plants have evolved complicated mechanism(s) at multiple levels to adapt to environmental stresses, including chilling stress. On one hand, stress-responsive signaling pathways regulate the expression of several downstream stress-related genes in response to abiotic stresses. For instance, *DREB1*/*CBF* belongs to the AP2/ERF superfamily transcription factors. The overexpression of *CBF1* and *CBF3* strongly induces the expression of *COR* (cold regulated) genes, thus increasing the tolerance of *Arabidopsis* to low-temperature conditions [5]. *PtrbHLH*, a citrus (*Poncirus trifoliata*) basic helix-loop-helix (bHLH) transcription factor, functions in chilling tolerance by positively regulating POD-mediated ROS scavenging [6]. On the other hand, non-enzymatic and enzymatic antioxidant systems participate in ROS scavenging to protect plant cells from oxidative damage [7]. Enzymatic antioxidants include superoxide dismutase (SOD), guaiacol peroxidase (POD), ascorbate peroxidase (APX), and catalase (CAT) [8]. SODs are responsible for the dismutation of O_2_^−^ to H_2_O_2_ and O_2_ and are considered the first defense against ROS. On the other hand, CAT, APX, and POD are enzymes that catalyze the conversion of H_2_O_2_ to H_2_O and O_2_ [9]. However, the mechanisms underlying the rice chilling stress response remain elusive, although a chilling-tolerance gene, *COLD1*, which encodes a trans-membrane protein that regulates G-protein-dependent low-temperature sensing and is required for the development of cold tolerance, has recently been cloned and functionally characterized in rice [10].

Many previous ‘-omics’ studies, which can reveal highly informative expression patterns of genes/proteins that reflect the differentiation of response among genotypes with contrasting stress tolerance, have been performed to unravel the abiotic stress-responsive mechanisms in plants [11–16]. A large proportion of identified DEGs/DEPs were annotated as photosynthetic genes/proteins or ribosomal constituents and were induced in response to environmental stresses. Six DEGs, including *RPS10*, *RPS11*, *RPL21*, *RPS23*, *RPL35a-3*, and a 60S acidic ribosomal protein encoding gene were identified and annotated as constituents of the large or small subunits of ribosomes [16]. All of them were repressed in response to early chilling stress. For the DEGs/DEPs associated with photosynthesis, 13 chlorophyll a/b-binding proteins and 10 chloroplast precursor DEGs were found to be down-regulated in rice under chilling stress [17]. Moreover, similar cases have been reported in drought and salt stress studies. For instance, chlorophyll synthetic and binding proteins, including the chlorophyll a/b-binding protein CP24, CHLH, CHLI, and others functioning in photosynthetic pathways, are significantly down-regulated in rice under drought stress [18, 19]. In a salt stress study, the levels of the chlorophyll a/b-binding proteins LHCA1, LHCA2, and LHCA4, were also found to be reduced under salt treatment [11]. Therefore, the comparative transcriptomic and proteomic profiling of genes/proteins provides new insights into environmental stress responses in plants.

Proteomic analysis is regarded as an effective strategy for the large-scale screening and identification of proteins. iTRAQ is a robust mass spectrometry technology that allows the quantitative comparison of protein abundance by measuring the peak intensities of reporter ions released from iTRAQ-tagged peptides by fragmentation during MS/MS [20]. iTRAQ-based proteomics has been widely applied in stress tolerance studies in plants [11, 21–24].

Here, a chilling-tolerant chromosome segment substitution line (CSSL), DC90, was developed by introgression of the genome of DP15 into the recurrent parent 9311 (chilling sensitive) in our previous study [16]. Therefore, the genetic background of DC90 is highly similar to that of 9311. Previous research has demonstrated that a major QTL harbored in the DP15 genomic segment of chromosome 12 accounted for the chilling-tolerance phenotype of DC90. We designated it as *CTS-12*. In this study, iTRAQ-based quantitative proteomics was performed to elucidate the effects of *CTS-12* on the protein levels of DC90 and 9311 under severe chilling stress and to identify chilling tolerance-associated DEPs by comparison with its recurrent parent. By this approach, we aimed to infer the potential mechanisms that are relevant to the chilling stress tolerance of wild rice.

## Methods

### Plant materials, growth, and treatment conditions

**Fig. 1:**
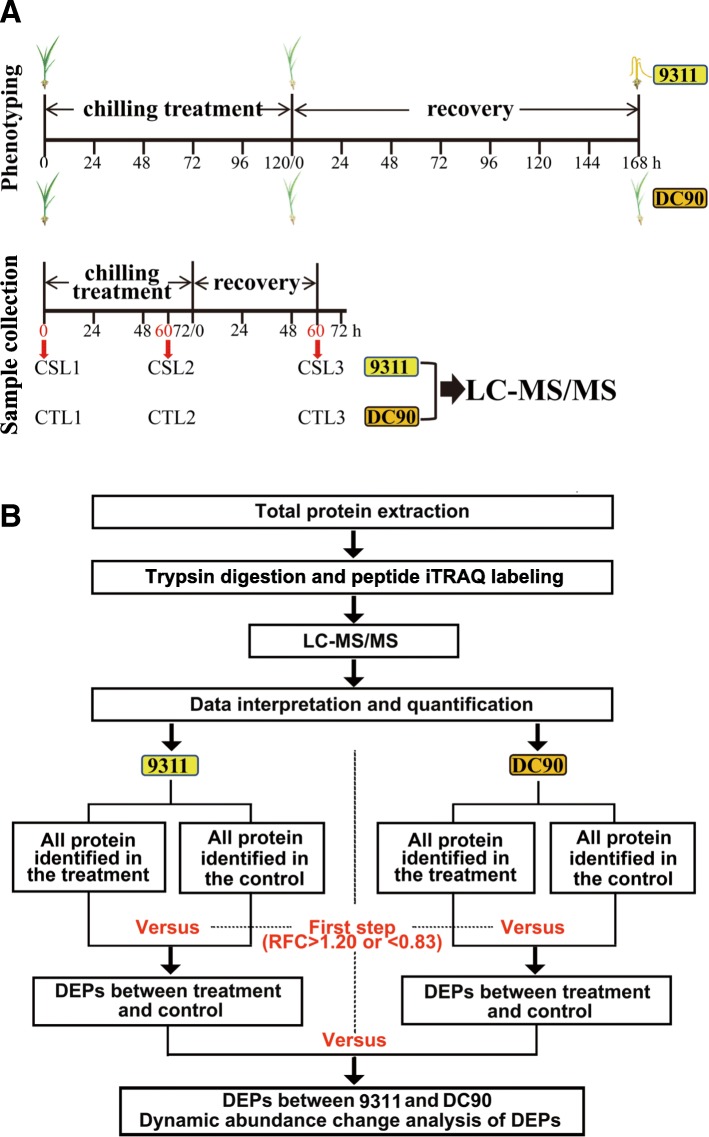
Schematic diagram showing the strategy of setting timepoints for phenotyping and sample collecting and the strategy for the comparative proteomic analysis of DC90 and 9311. **a**, Timepoint setting for chilling-tolerant phenotyping and for sample collection for LC-MS/MS; **b**, Strategy for the comparative proteomic analysis. CSL designates the chilling- and recovery-treated samples of 9311. CTL designates the chilling- and recovery-treated samples of DC90; CSL1, CSL2, and CSL3 represent the 0-h, 60-h chilling-treated, and 60-h recovery-treated samples of 9311. CTL1, CTL2, and CTL3 represent the 0-h, 60-h chilling-treated, and 60-h recovery-treated samples of DC90, respectively. RFC designates the relative fold change of DEPs between a treatment and its corresponding control

The collection of wild rice was done by Rongbai Li and deposited in wild rice resources conservation field of Guangxi University complying with legislation of China. The CSSL DC90 were then developed by crossing Guangxi common wild rice with 9311 to obtain chilling tolerance *indica* cultivar by Prof. Li in our previous breeding project. The growth of rice seedlings and chilling-stress phenotyping were performed according to previously described methods [16]. In brief, rice seeds were sowed in a plastic container filled with paddy soil and grown under natural conditions with natural sunlight to the three-leaf stage. Rice seedlings were then exposed to 10/8 °C (day/night) for 5 days with a photoperiod of day-13 h/night-11 h by supplementation with artificial light (20,000 Lux and 65% humidity). After chilling treatment, the rice seedlings were allowed to recover at 28/26 °C (day/night) for 7 days. Hydroponic culture was performed to grow rice plants for proteomic analysis as previously described [25]. Briefly, DC90 and 9311 plants were grown in 96-well PCR plates in a growth chamber with rice nutrient solution. Since 9311 is chilling sensitive and is unable to survive from 4-day (96 h) chilling treatment (Additional file 1). To study the dynamic changes of the proteins in response to chilling stress and recovery treatment, the following optimized treatment time lengths were used for sampling. The treatment time length, consisted of chilling treatment for 72 h followed by recovery for 60 h. Three replicates of whole-plant samples were harvested at 0 h, 60 h of chilling treatment, and 60 h of recovery. The DC90 samples from the three timepoints were designated CTL1, CTL2, and CTL3, which represented 0 h, 60 h of chilling treatment, and 60 h of recovery, respectively. Meanwhile, CSL1, CSL2, and CSL3 represented the 9311 samples at 0 h, 60 h of chilling treatment, and 60 h of recovery (Fig. 1a). All samples were frozen immediately in liquid nitrogen and stored at − 80 °C for total protein extraction.

### The determination of chilling stress induced histochemical and physiological changes in leaf tissues

Staining with 3,3′-Diaminobenzidine (DAB) was used to detect H_2_O_2_. The second leaves of three-leaf stage seedlings were cut into pieces and then subjected to vacuum infiltration in DAB solution (1 mg/mL in water, pH 5.8) for 10 min. The infiltrated samples were incubated for 8 h in the dark. Next, the chlorophyll in the samples was removed by boiling in absolute ethanol. The samples were observed and images taken under a stereo-microscope [26]. For the detection of O^2−^, leaf pieces were vacuum-infiltrated as described above in 0.1% nitroblue tetrazolium (NBT) with 0.1% Triton X-100 in 10 mM of phosphate buffer (pH 7.2) for 10 min. Chlorophyll was removed, and the samples were observed under a stereo-microscope [27].

Samples (0.5 g) were ground in liquid nitrogen to a fine powder and then resuspended with 10 mL of 50 mM PBS (pH 7.8) containing 5% PVP (*w*/*v*), 1 mM DTT, 0.1% Triton X-100 (*w/v*) and 0.1 mM EDTA at 4 °C. After centrifugation at 15000 *g* for 30 min, the supernatant was used for enzyme activity assays. The activity of CAT, POD, SOD, and APX were measured by the method as described [28]. The quantitation of relative electrolyte leakage and MDA were conducted following the methods as published previously [16, 28]. The AsA assay was performed by the method as described [29] with some necessary modifications. Rice seedlings were hydroponically cultured in 96-well PCR plates to the three-leaf stage, AsA (2 mM) was applied to the nutrient solution, and the seedlings were then exposed to 10/8 °C (day/night) for 5 days with a photoperiod of day-13 h/night-11 h.

### Protein extraction, trypsin digestion, and peptide desalting

The samples (1 g) were ground in liquid nitrogen into fine power and then resuspended in lysis buffer (8 M urea, 2 mM EDTA, 10 mM DTT and 1% Protease inhibitor cocktail). The homogenates were centrifuged at 13000 *g* and 4 °C for 10 min. The supernatant was precipitated with acetone at − 20 °C for 3 h. After centrifugation, the protein pellet was re-dissolved by urea buffer, which contained 8 M urea and 100 mM tetraethylammonium bromide (TEAB). The protein concentration was determined by using the Bradford Protein Assay Kit according to the manufacturer’s instructions.

Trypsin digestion and iTRAQ labeling were performed according to the method described in Mu et al. [15] with minor modification. First, 50 μg of protein was reduced with 10 mM DTT at 37 °C for 60 min and then alkylated with 25 mM iodoacetamide (IAM) at room temperature for 30 min in the dark. TEAB (100 mM) was added to dilute the protein sample to a urea concentration of less than 2 M. The protein samples were then digested with modified trypsin (sequencing grade) (protein:trypsin = 50:1) at 37 °C overnight. A second digestion was performed with protein:trypsin = 100:1 for 4 h.

After trypsin digestion, the peptides were desalted by a Strata X SPE column and vacuum dried. The peptides were then labeled with the iTRAQ 8-plex kit (AB Sciex, Foster City, CA, USA) according to the manufacturer’s instructions. The peptides from samples CSL1, CSL2, CSL3, CTL1, CTL2, and CTL3 were labeled with tags 115, 116, 117, 118, 119, and 121, respectively. All labeled samples were multiplexed and vacuum dried.

### High-resolution LC-MS/MS analysis

The labeled samples were reconstituted with HPLC solution A (2% ACN, pH 10) and then fractionated by high pH reverse-phase HPLC with a Waters Bridge Peptide BEH C18 (130 Å, 3.5 μm, 4.6 × 250 mm) column. The reconstituted samples were finally vacuum dried and then dissolved in 0.1% formic acid (FA, Fluka).

The analysis of LC-MS/MS was performed by Nano LC 1000 LC-MS/MS with a Proxeon EASY-nLC 1000 coupled to a Thermo Fisher Q Exactive. Trypsin-digested fractions were directly loaded onto a reversed-phase pre-column (Acclaim PepMap®100 C18, 3 μm, 100 Å, 75 μm × 2 cm) at 5 μL/min in 100% solvent A (0.1 M acetic acid in water). Next, the peptide eluents were loaded onto a reversed-phase analytical column (Acclaim PepMap® RSLC C18, 2 μm, 100 Å, 50 μm × 15 cm). The solvent B gradient (0.1% FA in 98% ACN) increased from 15 to 35% for 45 min, from 35 to 98% for 5 min, and finally was maintained at 98% for 5 min at a constant flow rate of 300 nL/min on an EASY-nLC 1000 system. The eluent was sprayed via the NSI source at an electrospray voltage of 1.9 kV and then analyzed by tandem mass spectrometry in Q Exactive. Full-scan MS spectra (from m/z 350 to 1800) were acquired in the Orbitrap with a resolution of 70,000, and ion fragments were detected at a resolution of 17,500. The 20 most intense precursors were selected for subsequent analysis. Tree-based ion trap HCD fragmentation was performed at a collision energy of 30% in the MS survey scan with 45.0 s dynamic exclusion.

### Protein identification and quantification

The LC-MS/MS raw data were searched against the UniProt *Oryza sativa* Japonica Proteome Database with the software Proteome Discoverer (version 1.3, Thermo Scientific). Trypsin was chosen as the enzyme, and two missed cleavages were allowed. Acetylation in N-Term was set as a variable modification and carbamidomethylation (C) as a fixed modification and oxidation. The searches were performed with a peptide mass tolerance of 20 ppm and a product ion tolerance of 0.02 Da with a 1% FDR. The iTRAQ 8-plex was chosen for quantification during the search. Before data exportation, the results were filtered with *p* < 0.05 (both the significance threshold and the ion score or expected cutoff).

### Criterion for DEP detection

To identify statistically significant DEPs, the relative fold change (RFC) of proteins was determined by the ratio in the treated samples and their corresponding untreated controls according to a previously described method [24]. The RFC of proteins in response to chilling treatment was calculated as the ratio of CSL2/CSL1 (9311) or CTL2/CTL1 (DC90), while the RFC in response to recovery was determined by the ratio of CSL3/CSL1 (9311) or CTL3/CTL1 (DC90) (Fig. 1). The proteins with RFC ≥ 1.200 (*p* < 0.05) were considered up-regulated, and those with RFC ≤ 0.833 (*p* < 0.05) were considered down-regulated.

### Protein hierarchical cluster analysis

Hierarchical cluster analysis was conducted on the quantified proteins from the biological replicates to assess the reproducibility of the MS data using a previously described method [30]. The relative abundance values of the identified proteins were subjected to Log2 normalization. Heatmaps and dendrograms were generated by pHEATMAP (R package) and g-plots [30], respectively. All the statistical analyses were performed in the R environment.

### Quantitative real time reverse transcription PCR analysis

Total RNA was isolated from three additional sets of samples (three biological replicates per sample). cDNA synthesis was performed by reverse transcription (RT) with the Thermo Scientific RevertAid First Strand cDNA Synthesis Kit (Cat# K1622) according to the manufacturer’s protocol. The sequences of DEP-encoding genes were downloaded from RGAP [31]. Primers were designed for qPCR to detect the relative expression of the selected DEPs at the transcript level, and PCR was performed by a Roche Lightcycler 480 Real-Time PCR System in 10 μL reactions with the SYBR Green PCR Master Mix kit (BIORAD, USA), following the manufacturer’s protocol. The relative expression of each gene was calculated according to the 2^-△△CT^ method [32]. The *GAPDH* gene (LOC_Os04g40950) was used as an endogenous reference for qPCR.

### GO enrichment analysis, functional classification, and expression profile analysis

The GO enrichment (http://geneontology.org) analysis was performed to annotate the DEPs under the categories of biological process (BP), cellular component (CC), and molecular function (MF) based on the GO-slim annotation database with Bonferroni correction by PANTHER (http://pantherdb.org) [33]. DAVID was used to perform functional clustering of the DEPs with EASE score = 0.05/high-stringency clustering algorithms according to the user manuals of the online tools (https://david.ncifcrf.gov/home.jsp) [34, 35].

Expression profile analysis was performed with the Short Time-series Expression Miner (STEM) clustering algorithm [36] to obtain an overview of the expression changes in DEPs in response to chilling stress. In brief, the expression data of the DEPs was subjected to Log2 transformation, and the Gramene *Oryza sativa* gene annotation file (http://www.geneontology.org/page/download-annotations) was used as a reference. The STEM clustering method was applied in the analysis. All the other fields were set to the defaults, and the following customized settings were used: the ‘Minimum Absolute Expression Change’ of the ‘Filtering’ option was set to 0.02, the ‘Correction Method’ was ‘Bonferroni Correction’, the ‘Permutation Test Should Permute Time Point 0’ was checked in the ‘Model Profiles’ option, and the ‘Minimum Correlation’ was set to 0.6 in the ‘Clustering Profiles’ option.

## Results

### Contrasting stress phenotypes of DC90 and 9311 in response to chilling treatment

**Fig. 2:**
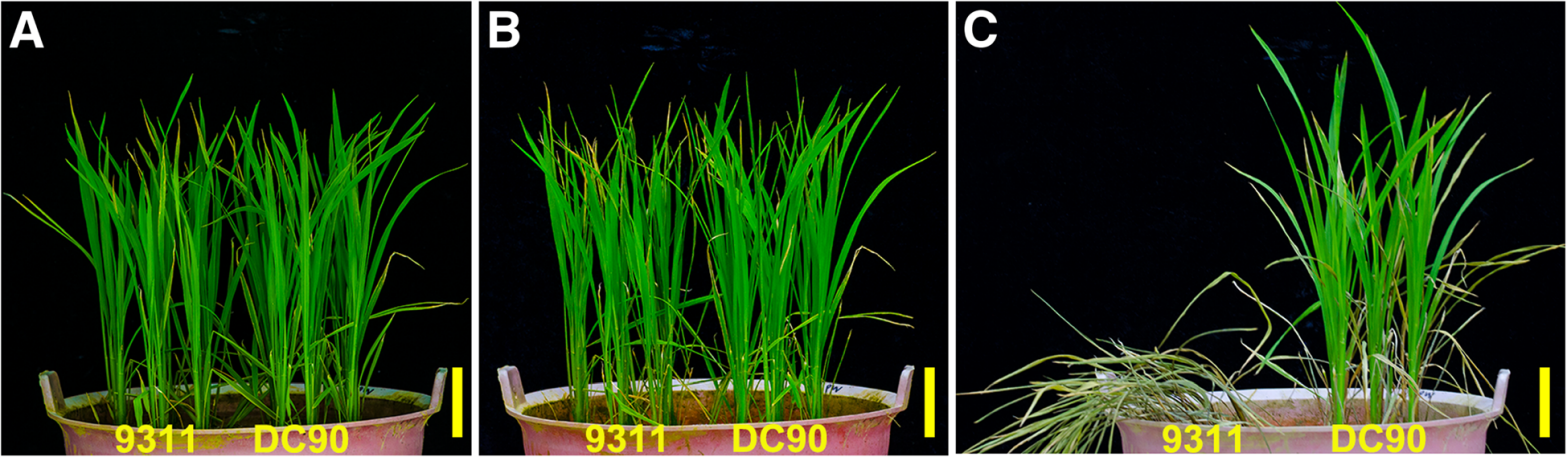
Chilling stress phenotypes of DC90 and 9311 at the seedling stage. **a**, The DC90 and 9311 seedlings before exposure to low temperature; **b**, The DC90 and 9311 seedlings after 5-day chilling treatment; **c**, The DC90 and 9311 seedlings after 7-day recovery treatment. Scale bars = 5 cm in **a**, **b**, and **c**

After 5 days chilling stress treatment, no obvious difference between DC90 and 9311 was visible (Fig. 2a, b). During the recovery period, phenotypic differences between DC90 and 9311 were observed to develop gradually (Fig. 2c). By the end of recovery, the 9311 seedlings were completely wilted, whereas the DC90 seedlings were able to survive the chilling stress, as reported previously [16]. Similar phenotypic differences can be obtained under hydroponic culture conditions (Additional file 2).

To understand how *CTS-12* mediated the mechanisms underlying chilling stress responses at the proteomic level, hydroponically cultured rice seedlings were subjected to chilling stress and recovery following an optimal treatment scheme (Fig. 1a). The treated samples were subjected to proteomic analysis.

### Identification and quantitation of proteins by iTRAQ-based LC-MS/MS analysis

By using iTRAQ labeling LC-MS/MS analysis, a total of 4388 proteins were specifically identified from 1,178,076 LC-MS/MS spectra and 19,405 peptides in three independent experiments with a 1% FDR (false discovery rate) (Additional file 3; Additional files 1, 2, 3, 4; Additional file 5). Among these results, 2819 proteins were quantified in all replicates of all samples (Additional files 2, 3, 4).

**Fig. 3:**
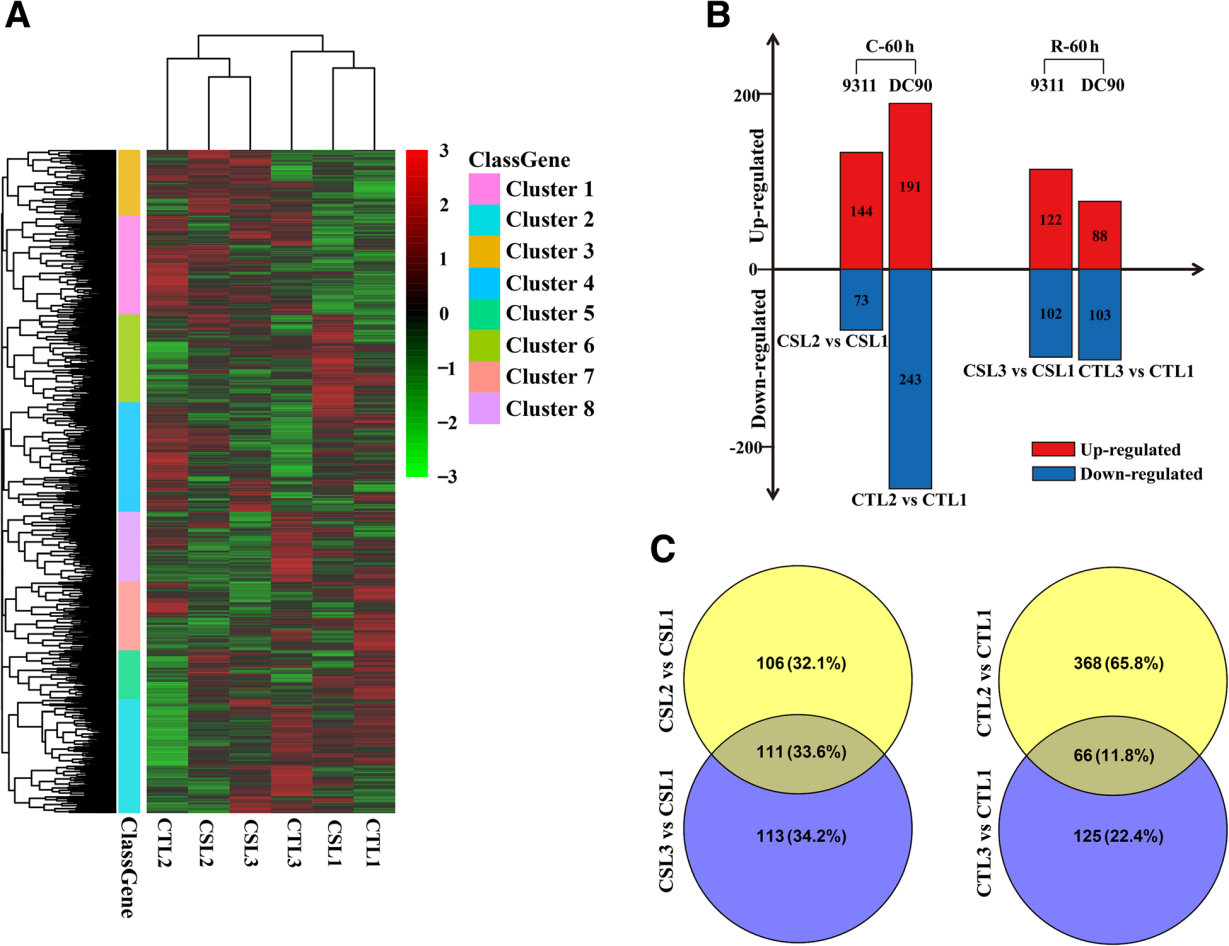
Hierarchical clustering of quantified proteins and DEPs identified in chilling- and recovery-treated DC90 and 9311. **a**, Hierarchical clustering of quantified proteins based on three replicates of LC-MS/MS data; **b**, DEPs identified in chilling- and recovery-treated DC90 and 9311 by comparison with their corresponding controls; **c**, Venn diagram showing common and specific DEPs identified in the chilling- and recovery-treated samples of 9311 and DC90, respectively. CSL1, CSL2, and CSL3 represent the 0-h, 60-h chilling-treated, and 60-h recovery-treated samples of 9311, and CTL1, CTL2, and CTL3 represent the 0-h, 60-h chilling-treated, and 60-h recovery-treated samples of DC90, respectively. C-60 h and R-60 h indicate the chilling- and recovery-treated stages, respectively

Hierarchical clustering was conducted to acquire a comprehensive overview of the expression profiles of these proteins by using the pHEATMAP package in R. The results showed that the three replicates of each timepoint of 9311 and DC90 were clustered into the same respective clades (Additional file 6), indicating the reproducibility and reliability of our LC-MS/MS data. Notably, clustering based on mean of protein abundance revealed clear differences among the three timepoints (Fig. 3a), suggesting that the rice plants mobilized numerous proteins and differentially regulated their abundance to cope with chilling stress.

### Identification of statistically significant DEPs

Student’s *t*-test was applied to determine if the proteins in the untreated and treated samples were significantly different based on 2819 quantified proteins (*p <* 0.05). According to the criteria RFC ≥ 1.200 or ≤ 0.833 and *p <* 0.05, a total of 217 DEPs (144 up−/73 down-regulated) and 434 DEPs (191 up−/243 down-regulated) were identified in seedlings of 9311 and DC90, respectively, under chilling stress treatment (Fig. 3b). More than twice as many DEPs were identified in DC90 as in 9311, providing further evidence that many proteins in DC90 were regulated in response to chilling stress. Under recovery treatment, a total of 224 DEPs (122 up−/102 down-regulated) and 191 (88 up−/103 down-regulated) were identified in 9311 and DC90, respectively (Fig. 3b). Thus, a higher total number of DEPs were identified in DC90 than in 9311 (Fig. 3b, c; Additional files 3, 4, 5, 6). Among the 330 DEPs in 9311, 106 (32.10%) and 113 (34.20%) were specifically identified during the periods of chilling and recovery, respectively. One hundred and eleven (33.60%) were shared by both stages (Fig. 3c). However, in DC90, only 66 out of 559 DEPs (11.80%) were common to both stages, while 65.80% of DEPs (368 out of 559) were specifically identified in the chilling treatment stage (Fig. 3c). These results further supported the notion that different groups of proteins were mobilized in response to the two treatment stages in the chilling-tolerant genotype.

To compare the responsive DEPs between two genotypes during the whole treatment period, the following strategy was used to select statistically significant DEPs between DC90 and 9311 for further comparative analysis. First, all proteins with *p* < 0.05 in CSL2/CSL1, CSL3/CSL1, CTL2/CTL1, and CTL3/CTL1 were selected to obtain 4 protein groups, which included up-regulated (RFC ≥ 1.200), down-regulated (RFC ≤ 0.833), and NA (0.833 < RFC < 1.200) subsets (Additional files 3, 4, 5, 6). Second, the datasets for CSL2/CSL1 and CSL3/CSL1, CTL2/CTL1 and CTL3/CTL1, including their RFC data, were respectively combined to obtain the protein groups. The proteins with 0.833 < RFC < 1.200 in both stages and the proteins with *p* < 0.05 in only one treated stage were eliminated from each group (Additional files 4, 5, 6, 7, 8). Finally, totals of 206 and 155 were statistically significant DEPs were selected in 9311 and DC90, respectively. Further GO enrichment analysis, functional classification, and expression profile analysis were based on these two lists of proteins.

### Validation of gene expression of differentially expressed proteins

**Fig. 4:**
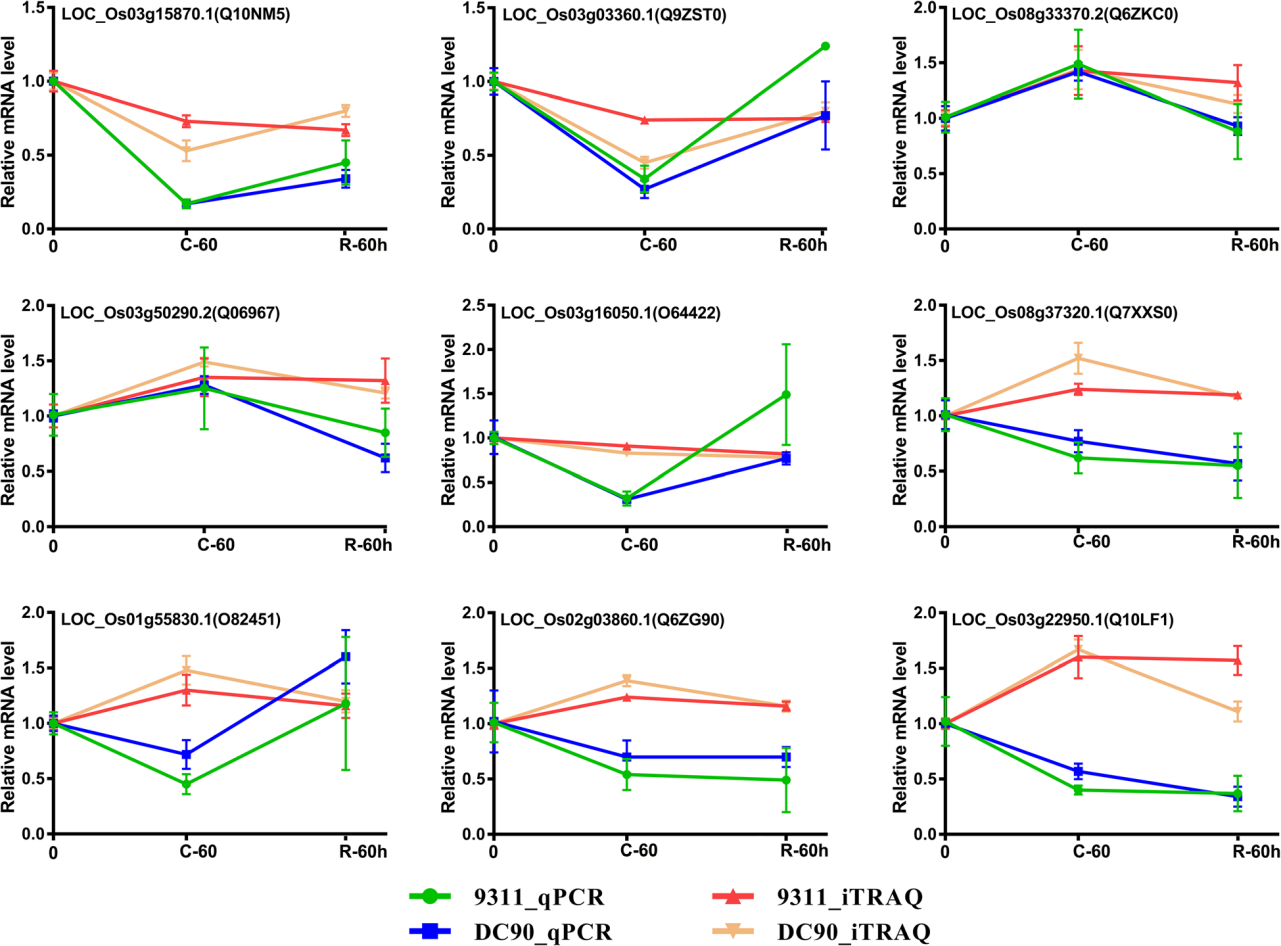
Comparative analysis of the protein and mRNA profiles of 9 representative DEPs. The X-axis represents the timepoints in the chilling and recovery treatments. The Y-axis indicates the normalized relative mRNA and protein levels. The green and blue lines represent the patterns of mRNA expression in 9311 and DC90, and the red and orange lines represent the patterns of protein expression in 9311 and DC90, respectively

Nine common DEPs were randomly selected for qPCR analysis to test the correlation of expression profiles between proteins and their corresponding mRNAs (Additional file 7). We compared the mRNA levels after 60-h chilling and 60-h recovery treatment with the iTRAQ data. The qPCR results showed that the expression profiles of 4 genes, LOC_Os03g15870.1, LOC_Os03g03360.1, LOC_Os08g33370.2, and LOC_Os03g50290.2, were in agreement with the iTRAQ data, whereas 5 genes (LOC_Os03g16050.1, LOC_Os08g37320.1, LOC_Os01g55830.1, LOC_Os02g03860.1, LOC_Os03g22950.1) displayed wholly different expression patterns from those observed by the iTRAQ analysis (Fig. 4). The inconsistent expression profiles between qPCR and iTRAQ indicated the transcriptional level of genes were not always parallel to their corresponding protein level [37].

### GO enrichment analysis and functional classification of DEPs

**Fig. 5:**
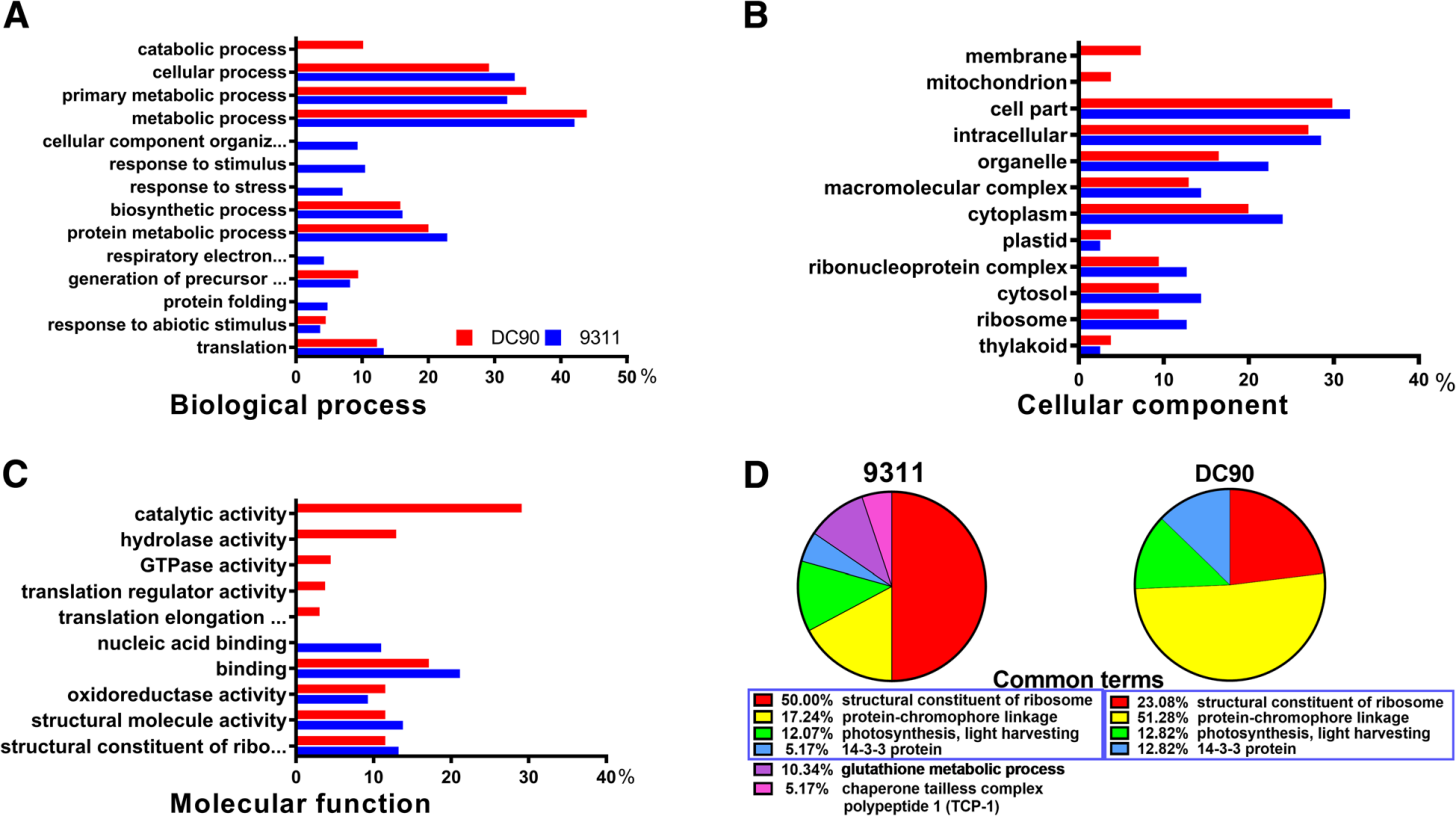
GO enrichment and functional classification analysis of DEPs between DC90 and 9311 during the whole period of chilling and recovery treatment. **a**, Biological process GO terms with DEPs enriched in DC90 and 9311; **b**, Cellular component GO terms with DEPs enriched in DC90 and 9311; **c**, Molecular function GO terms with DEPs enriched in DC90 and 9311; **d**, Functional classification of DEPs identified in DC90 and 9311

To further understand the functions of the identified DEPs, GO analysis and functional classification were performed. In the GO analysis, 177 and 142 protein IDs in 9311 and DC90, respectively, were mapped to the PANTHER database [33] (Additional files 1, 2, 3, 4, 5, 6, 7, 8). The DEPs in both genotypes were significantly enriched in 14 biological processes, 12 cellular components, and 10 molecular function subgroups. Within the biological process subgroup, the cellular process, metabolic process, and primary metabolic process groups were prominent, indicating that the primary metabolic processes are easily affected in response to chilling stress (Fig. 5a; Additional files 1, 2, 3, 4, 5, 6, 7, 8). The cell part, intracellular, organelle, and cytoplasm categories were four cellular compartments in which DEPs were highly localized (Fig. 5b; Additional files 1, 2, 3, 4, 5, 6, 7, 8). In terms of molecular function, the DEPs from both genotypes were highly enriched in the same or similar sets of terms, such as binding, oxidoreductase activity, structural constituent of ribosome, structural molecule activity, and nucleic acid binding. However, translation elongation factor activity, translation regulator activity, GTPase activity, hydrolase activity, and catalytic activity were specifically represented in DC90 (Fig. 5c; Additional files 1, 2, 3, 4, 5, 6, 7, 8). Strikingly, the majority of over-represented cellular component GO terms were common to both genotypes. Furthermore, the identified DEPs consisted specifically of cytosol, ribosome, and plastid components (Fig. 5b). The functional clustering of DEPs by using DAVID gave results consistent with these findings (Fig. 5d). Among the over-represented functional categories, the structural constituent of ribosome, protein-chromophore linkage, photosynthesis, light harvesting, and 14–3-3 protein groups were shared by both 9311 and DC90 (Fig. 5d).

Taken together, our results indicated that chilling stress-responsive proteins were mainly localized in the cytosol, ribosomes, and plastids/chloroplasts and that they might play vital roles in chilling stress-related responses.

### Distinctly different dynamic changes of enriched chloroplastic DEPs in two genotypes during the period of chilling and subsequent recovery

**Fig. 6:**
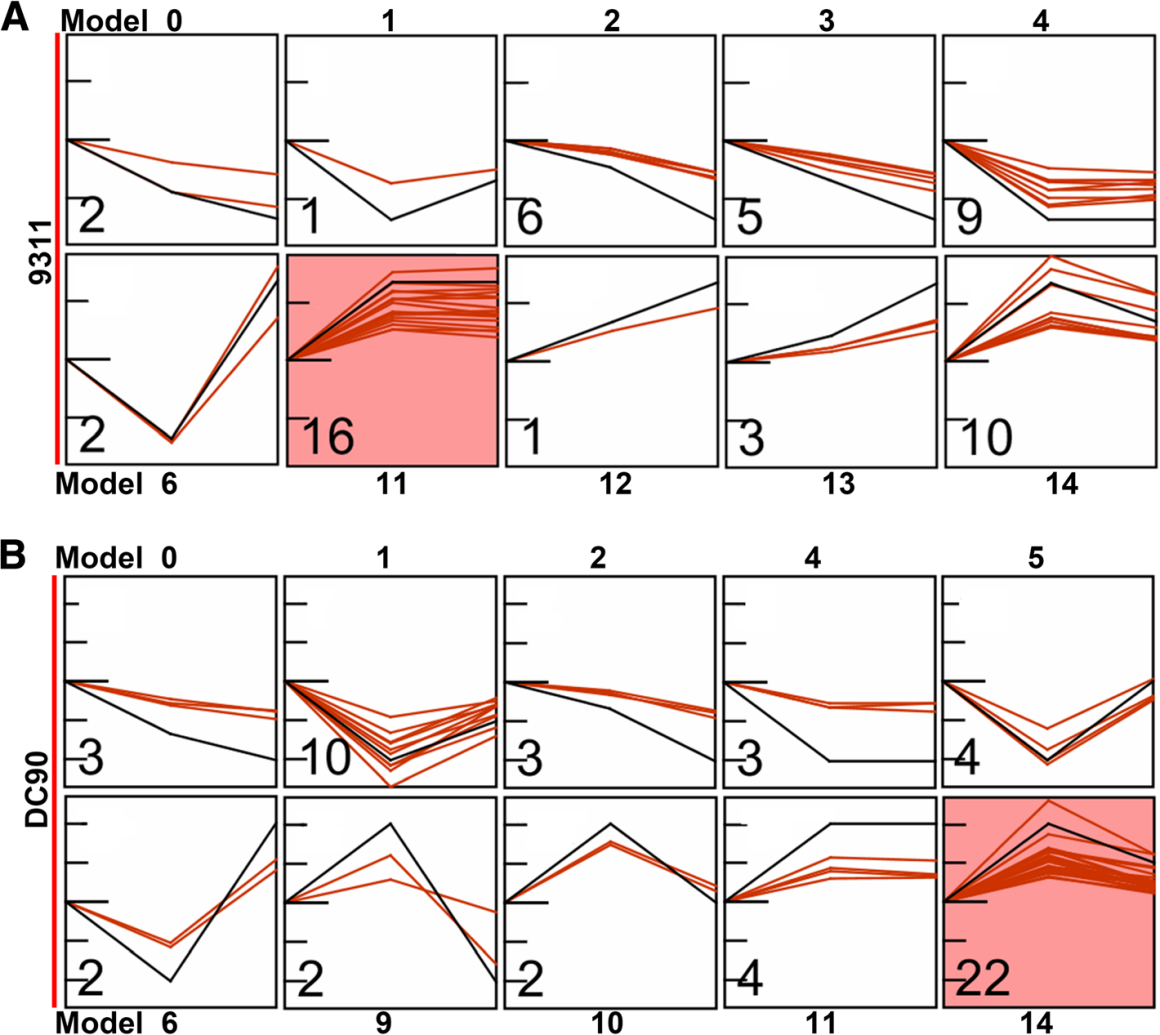
Profile model analysis of common DEPs during the whole period of chilling and recovery treatment. **a**, Profile model analysis based on the expression data of 9311 DEPs; **b**, Profile model analysis based on the expression data of DC90 DEPs. The number at the bottom-left corner represents the number of DEPs assigned to the corresponding model. Colored profiles indicate a statistically significant number of genes assigned to that category. *P* < 0.05 was set as the significance level with Bonferroni correction

To gain deeper insight into the dynamic changes in proteins between DC90 and 9311 during the whole period of chilling and recovery, we clustered the DEPs based on their abundance data using the STEM clustering algorithm [38]. The results showed that all the DEPs of DC90 and 9311 were clustered into 16 profile models (Additional file 9; Additional files 1, 2, 3, 4, 5, 6, 7, 8, 9, 10). The query lists of the two genotypes had 55 DEPs in common, but only 13 (23.64%) had the same profiles (Additional files 1, 2, 3, 4, 5, 6, 7, 8, 9, 10). Among these 55 DEPs, interestingly, 73.36% of which in 9311 either displayed a continuous increase (models 12 and 13) or decrease (models 0, 2, and 3) in abundance over the time period studied or retained the chilling-induced abundance during the subsequent recovery (models 4 and 11). However, in DC90, only 23.62% displayed such profiles (Tables 1, 2). The majority of DC90 proteins (76.34%) were enriched in 6 profile models (1, 5, 6, 9, 10, and 14) in which the abundance changes of individual proteins displayed a trough or a peak (Table 1; Fig. 6a, b; Additional files 1, 2, 3, 4, 5, 6, 7, 8, 9, 10). These differences were also clear in the groups of DEPs that were specifically identified in either DC90 or 9311. For instance, only 31.13% (47/151) of proteins in 9311 showed a trough or a peak, whereas 58.00% (58/100) of proteins in DC90 showed the same tendency over the time period studied (Additional files 2, 3, 4, 5, 6, 7, 8, 9, 10).

**Table 1:** Summary of the expression profiling of DEPs common to DC90 and 9311 by using STEM

Profile model	9311		DC90	
	DEP amount	Percent (%)	DEP amount	Percent (%)
0	2	3.64	3	5.45
1	1	1.82	10	18.18
2	6	10.91	3	5.45
3	5	9.09	0	0.00
4	9	16.36	3	5.45
5	0	0.00	4	7.24
6	2	3.64	2	3.64
7	0	0.00	0	0.00
8	0	0.00	0	0.00
9	0	0.00	2	3.64
10	0	0.00	2	3.64
11	16	29.09	4	7.27
12	1	1.82	0	0.00
13	3	5.45	0	0.00
14	10	18.18	22	40.00
15	0	0.00	0	0.00

**Table 2:** Model profiling of common DEPs identified during the chilling and recovery treatment periods of 9311 and DC90

UniProt_ID	Gene ID/Name		9311				DC90			Subcellular location		Putative function
		0 h	C^a^-60 h	R^b^-60 h	PM^c^	0 h	C^a^-60 h	R^b^-60 h	PM^c^	CP^d^	UniP^e^	
Q10NM5	LOC_Os03g15870	0	−0.46	−0.58	0	0	− 0.93	− 0.32	1	Y	–	50S ribosomal protein L4, chloroplast, putative
Q2QN11	LOC_Os12g39360	0	−0.2	− 0.3	0	0	−0.33	− 0.27	4	–	–	Eukaryotic aspartyl protease family protein
Q650W6	Os09g0565200	0	−0.12	−0.32	2	0	−0.31	−0.48	0	Y	–	Similar to nucleic acid-binding protein precursor
Q7X8A1	Os04g0459500	0	−0.11	−0.31	2	0	−0.33	−0.38	4	Y	–	Glyceraldehyde-3-phosphate dehydrogenase
Q0IWS0	Os10g0492300	0	−0.1	− 0.27	2	0	−0.27	− 0.27	4	Y	–	Similar to IAP100
Q7X7H3	Os04g0490800	0	−0.12	−0.28	3	0	−0.23	−0.38	0	Y	–	OSJNBa0076N16.12 protein
O64422	Os03g0267300	0	−0.13	−0.28	3	0	−0.27	−0.37	0	Y	C	Fructose-1,6-bisphosphatase, chloroplastic
Q943K1	Os01g0869800	0	−0.25	−0.43	3	0	−1.07	−0.59	1	Y	–	22-kDa photosystem II protein, photoprotection
Q8S3R2	Os02g0707100	0	−0.17	− 0.32	3	0	−0.45	− 0.25	1	–	–	Similar to monodehydroascorbate reductase, seedling isozyme (EC 1.6.5.4) (MDAR seedling)
Q0DGH0	Os05g0533100	0	−0.19	−0.36	3	0	−0.6	0.03	5	–	–	Similar to plasminogen activator inhibitor 1 RNA-binding protein
P0C367	psbC	0	−0.34	−0.34	4	0	−0.66	−0.32	1	–	C	Photosystem II CP43 reaction center protein
P0C364	psbB	0	−0.35	−0.34	4	0	−0.88	−0.43	1	–	C	Photosystem II CP47 reaction center protein
P0C434	psbA	0	−0.35	−0.38	4	0	−1.08	−0.44	1	–	C	Photosystem II protein D1
A0A0N7KJ79	Os04g0473150	0	−0.57	− 0.51	4	0	−1.35	−0.7	1	–	–	Similar to photosystem II protein D1
Q8SAY0	RPL18	0	−0.42	−0.36	4	0	−0.8	−0.29	1	Y	C	50S ribosomal protein L18, chloroplastic
Q9ZST0	RPL5	0	−0.43	−0.42	4	0	−1.14	−0.32	1	Y	C	50S ribosomal protein L5, chloroplastic
Q9XJ28	rps9	0	−0.49	−0.5	4	0	−0.88	−0.19	5	Y	–	30S ribosomal protein S9, chloroplast, putative
Q6KA00	Os02g0822600	0	−0.56	−0.47	4	0	−1.06	−0.24	5	Y	–	Similar to 50S ribosomal protein L9
P0C355	psaA	0	−0.24	− 0.27	4	0	−1.01	−0.21	5	–	C	Photosystem I P700 chlorophyll a apoprotein A1
P12330	CAB1R	0	0.6	0.55	11	0	0.6	−0.79	9	Y	C	Chlorophyll a/b-binding protein 1, chloroplastic
Q10LF1	LOC_Os03g22950	0	0.67	0.64	11	0	0.74	0.15	10	Y	–	Acyl carrier protein
O22386	RPL12–2	0	0.37	0.34	11	0	0.67	0.23	14	Y	C	50S ribosomal protein L12, chloroplastic
Q6ZKC0	GF14C	0	0.5	0.39	11	0	0.52	0.18	14	–	Cyt	14–3-3-like protein GF14-C
Q06967	GF14F	0	0.42	0.38	11	0	0.57	0.28	14	–	Cyt	14–3-3-like protein GF14-F
Q10KY5	Os033g0366000	0	0.41	0.43	11	0	0.46	0.2	14	–	–	10 kDa chaperonin, putative, expressed
Q8H2U6	Os07g0661700	0	0.76	0.79	11	0	0.87	0.61	14	–	–	Conserved hypothetical protein
A0A0P0V9F2	Os01g0803200	0	0.59	0.61	11	0	0.67	0.46	14	–	–	Cysteine proteinase inhibitor (Fragment)
Q0JPA6	Os01g0233000	0	0.53	0.54	11	0	0.7	0.35	14	–	–	Salt stress root protein RS1
Q6K1Q5	Os02g0622400	0	0.53	0.45	11	0	0.42	0.27	14	–	–	Glycolipid transfer protein-like
Q07661	NDKR	0	0.52	0.59	11	0	0.64	0.44	14	–	–	Nucleoside diphosphate kinase 1
Q2QWN3	Os12g0189400	0	0.34	0.29	11	0	0.36	0.24	14	Y	–	Similar to photosystem I reaction center subunit N, chloroplast precursor (PSI- N)
Q7XXS0	Os08g0478200	0	0.31	0.25	11	0	0.6	0.22	14	–	M	ATP synthase subunit d, mitochondrial
Q8LNF2	Os10g0502000	0	0.27	0.2	11	0	0.44	0.13	14	Y	–	Similar to thylakoid lumenal 17.4 kDa protein
Q6ETK1	Os02g0180200	0	0.27	0.26	11	0	0.39	0.17	14	Y	–	Uncharacterized protein family UPF0133 domain-containing protein
Q6K623	Os02g0612900	0	0.26	0.46	12	0	0.4	0.33	11	–	–	Similar to temperature stress-induced lipocalin
Q0IZF1	Os09g0572700	0	0.13	0.35	13	0	0.32	0.14	14	–	–	Similar to blue copper-binding protein
Q2QND8	Os12g0569300	0	0.13	0.37	13	0	0.42	0.23	14	Y	–	Thaumatin, pathogenesis-related family protein
Q8S9Q6	Os01g0941200	0	0.09	0.27	13	0	0.32	0.11	14	–	–	Similar to glucan endo-1,3-beta-glucosidase GII precursor (EC 3.2.1.39)
A0A0P0X334	Os07g0176900	0	0.29	0.18	14	0	0.3	−0.12	9	Y	–	Similar to ribose-5-phosphate isomerase precursor
Q6YS11	Os08g0282400	0	0.42	0.29	14	0	0.79	0.21	10	–	–	Similar to alpha-SNAP (fragment)
Q6ESR4	P0684A08.9–1	0	0.37	0.2	14	0	0.3	0.32	11	–	–	Dehydration-stress inducible protein 1
Q5ZCK5	CML16	0	0.34	0.18	14	0	0.57	0.53	11	–	–	Probable calcium-binding protein CML16

Notes: ^a^ indicates chilling stress treatment; ^b^ indicates recovery treatment; ^c^ designates Profile Model of DEPs, obtained by using STEM; ^d^ designates the subcellular locations of DEPs predicted by using ChloroP; ^e^ designates information on the subcellular locations of DEPs annotated in the UniProt database

In the list of common proteins, two major groups of DEPs caught our attention (Table 2; Additional files 1, 2, 3, 4, 5, 6, 7, 8, 9, 10). The first group contained 23 proteins located in the chloroplasts, and either are core components of the photosystem I and II reaction centers, such as psbA (P0C434), psbC (P0C367), psbB (P0C364), Q2QWN3, and A0A0N7KJ79, or are involved in chlorophyll binding, light harvesting by photosystems I and II, and the photodamage repair of D1, such as psaA (P0C355), CAB1R (P12330), and Q943K1 (Table 2; Additional files 1, 2, 3, 4, 5, 6, 7, 8, 9, 10). The second group contained 6 proteins, Q10NM5, RPL18 (Q8SAY0), RPL5 (Q9ZST0), rps9 (Q9XJ28), Q6KA00, and RPL12–2 (O22386), that appeared to be constituent parts of the large and small subunits of chloroplastic ribosomes (Table 2; Additional files 1, 2, 3, 4, 5, 6, 7, 8, 9, 10). Intriguingly, these proteins displayed similar regulation directions during the chilling treatment stage in both genotypes, which manifested as cold acclimation in response to chilling stress (Fig. 6; Table 2; Additional files 1, 2, 3, 4, 5, 6, 7, 8, 9, 10). However, different regulation directions of these proteins were evident during recovery. For instance, psbA and RPL18 were clustered in profile model 4 in 9311, which retained a down-regulated low level of abundance, whereas their expression levels were clearly up-regulated (relative to the abundance at the 60-h chilling treatment timepoint) during the recovery in DC90 (model 1) and appeared the de-acclimation of chilling-induced abundance change of photosynthetic and ribosomal proteins, although they could not recover to the level of those proteins in the untreated control. Furthermore, both CAB1R and Os12g0189400 were clustered in profile model 11 in 9311, whereas in DC90, they were clustered in models 9 and 14, respectively, which showed the chilling-induced high abundance returning to the normal level during the recovery (Fig. 6; Table 2; Additional files 1, 2, 3, 4, 5, 6, 7, 8, 9, 10). Similar profiles were observed in specific protein groups identified in 9311 and DC90. Twenty-seven proteins in 9311 were annotated as constituent parts of ribosomes, 19 of which (70.37%, 19/27) were clustered in models 4 and 11. However, although fewer ribosomal proteins were annotated in DC90 than in 9311, 70.59% (12/17) displayed the patterns defined by models 1, 5, 6, 7, 8, 9, and 14 (Additional file 9; Additional files 2, 3, 4, 5, 6, 7, 8, 9, 10).

### Different H_2_O_2_ scavenging capabilities in DC90 and 9311

**Fig. 7:**
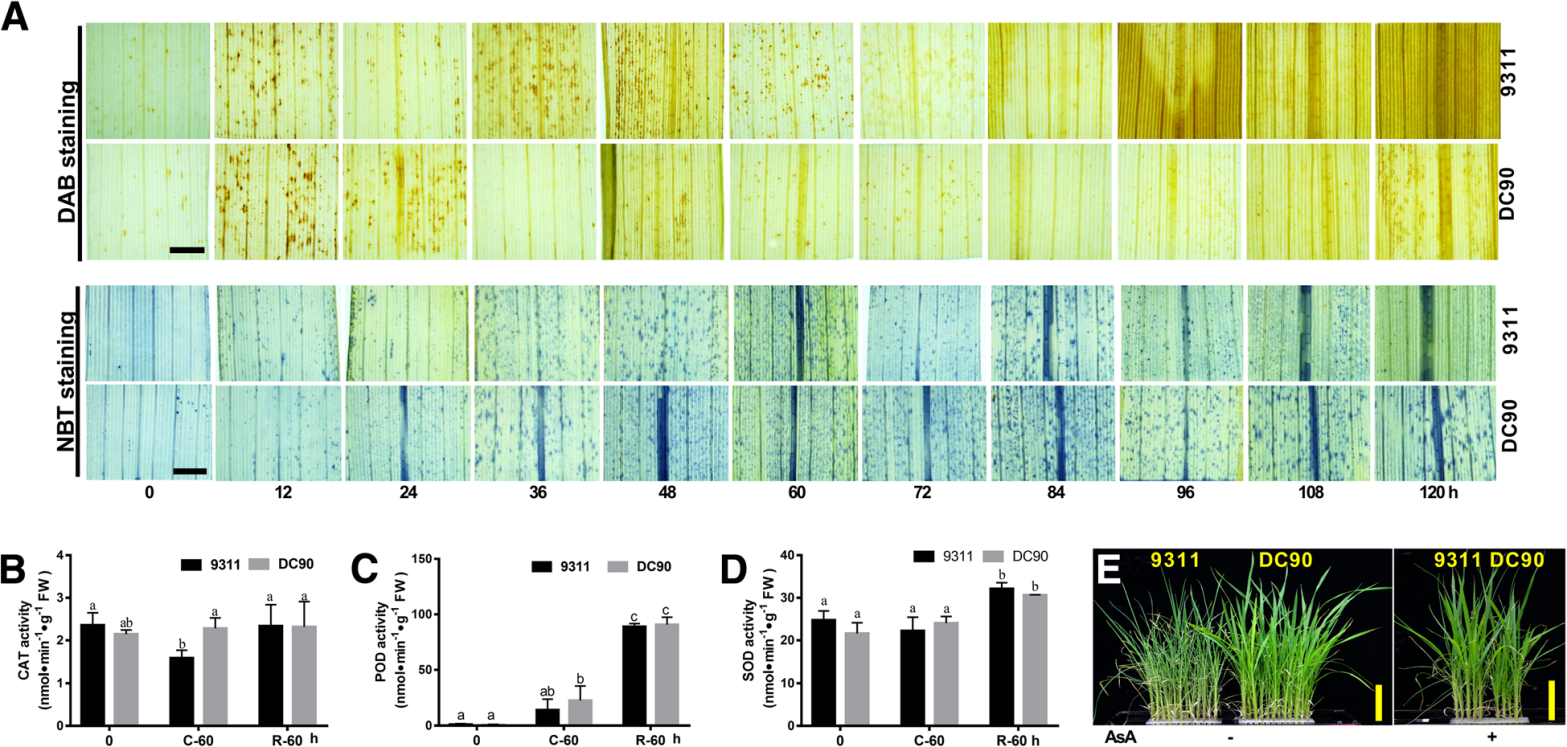
ROS production and elimination analysis of the samples of DC90 and 9311 under chilling and recovery treatment. **a**, DAB- and NBT-stained leaves of DC90 and 9311; **b**, CAT activities of DC90 and 9311 under chilling and recovery conditions; **c**, POD activities of DC90 and 9311 under chilling and recovery conditions; **d**, SOD activities of DC90 and 9311 under chilling and recovery conditions; **e**, Phenotypes of chilling-treated DC90 and 9311 plants with/without the exogenous application of ascorbic acid (AsA). For the staining assay, at least three independent experiments were performed, and representative images are shown. The different letters at the top of each column indicate statistically significant differences based on ANOVA and Tukey’s Duncan test (*P* < 0.05). Scale bar = 0.2 cm in A, B. Scale bar = 10 cm in D

Chilling stress induced ROS accumulation in plant [13]. Furthermore, three down-regulated ROS scavenging associated DEPs, prx11 (Q9FYP0), CATB (Q0D9C4), and catalase (Q10S82), were specifically identified in 9311 (Additional files 2, 3, 4, 5, 6, 7, 8, 9, 10). The enzymes catalyze the conversion of H_2_O_2_ to H_2_O and O_2_ [9]. To investigate the possible correlation of the accumulating ROS and dynamic changes in chloroplast photosynthetic and ribosomal proteins, ROS production and scavenging in the samples from the chilling and recovery stages were tested histochemically and physiologically. DAB and NBT staining showed that both DC90 and 9311 began to accumulate ROS at an early timepoint in the chilling treatment (12 h) (Fig. 7a). Beginning with the 36-h timepoint, the DAB-stained spots in DC90 gradually disappeared, whereas H_2_O_2_ continued to accumulate until 72 h in 9311, suggesting that different H_2_O_2_ scavenging capabilities in DC90 and 9311. This result was confirmed by the analysis of H_2_O_2_ and superoxide scavenging-related enzymes, and physiological changes in rice plants (Fig. 7b, c; Additional file 11 C, D, E). In line with proteomic results, the CAT activity was significantly inhibited after 60 h of chilling stress treatment by comparison with that of DC90 and might be a certain reason for the overproduction of H_2_O_2_ in 9311, although no difference was observed at the 0-h timepoint or after 60 h of recovery in either genotype. The activity of another H_2_O_2_-scavenging enzyme, POD, was induced at the 60-h chilling and 60-h recovery timepoints in both DC90 and 9311 by comparison with the untreated control, but no significant differences between genotypes were observed at either timepoint. The activity of SOD and APX showed very similar patterns in DC90 and 9311 at each timepoint (Fig. 7a, d; Additional file 11 E).

The results suggested that the overproduction of H_2_O_2_ under chilling stress might account for the chilling sensitivity phenotype of 9311. This idea was further supported by the exogenous application of ascorbic acid (AsA) to 9311 under chilling stress (Fig. 7e). AsA is an antioxidant reagent that participates in H_2_O_2_ scavenging in plant cells. The exogenous application of AsA rescued the chilling-induced cell death in 9311.

Taken these together, the abundances of both common and specific proteins identified in DC90 and 9311 exhibited completely different chilling- and recovery-induced regulation. The results also suggested that the *CTS*-12 might mediate the chilling-induced cold acclimation and de-acclimation of the abundance of photosynthetic and ribosomal proteins in the periods of chilling and recovery. Thereby conferred wild rice chilling tolerance under severe chilling stress. Meanwhile, the balance between H_2_O_2_ production and elimination in rice plants under chilling stress might play a certain role in biochemical cold acclimation and de-acclimation.

## Discussion

In this study, DC90 was used to dissect the mechanisms underlying the cold tolerance conferred by DP15 via comparative proteomic analysis. Two continuous treatment stages were designed to facilitate obtaining a global picture of the dynamic regulation performed during the transition between the chilling and recovery stages.

### Chilling-responsive downstream regulation in rice occurs mainly in chloroplasts

The chloroplast is an important organelle for photosynthesis in plant cells. However, recent studies showed that the chloroplast not only plays its universal role in photosynthesis but also participates in the response to environmental stresses [39, 40]. Specifically, photosynthetic genes/proteins were regulated in response to environmental stresses [11, 17, 18]. When rice plants were subjected to chilling, drought, or salt stresses, photosynthetic activity was greatly inhibited. Genes associated with chlorophyll biosynthesis, chlorophyll a/b-binding proteins, the light-harvesting complex, the PSII and PSI core complexes, and chloroplast precursors were significantly down-regulated [11, 17, 18]. Ribosomal proteins also perform extra functions in response to environmental stresses [16, 41–43]. For example, in soybean, chilling induced the expression of 3 ribosomal genes, *RPS6*, *RPS13*, and *RPL37* [44]. In rice, *34 RPLs* in tissues covering the major stages of rice growth were highly responsive to diverse stresses, indicating that ribosomal genes appear to play roles in stress amelioration in addition to housekeeping [45]. In this study, among 55 common DEPs, two major group proteins were highlighted. One consisted of 23 chloroplast-localized proteins, including core components of photosystems I and II, such as psbA, psbC, psbB, and PSI-N, as well as proteins involved in chlorophyll binding (CAB1R), light harvesting (psaA), and photoprotection (Q943K1). The other consisted of 6 chloroplastic ribosome constituents, Q10NM5, RPL18, RPL5, rps9, Q6KA00, and RPL12–2. STEM analysis showed that both groups of DEPs were significantly regulated in response to chilling and recovery but exhibit entirely different regulatory patterns in 9311 and DC90 (Fig. 6; Table 2; Additional files 9, 10). Thus, the chloroplast is inferred to be an important target organelle for downstream regulation in response to environmental stresses in wild rice.

### Rice adaptation to chilling stress by the proteomic regulation of cold acclimation and de-acclimation of chloroplastic proteins

Plant growth and distribution are affected by low-temperature stress. Overwintering plants can adapt to freezing temperature via the orchestrated expression of several sets of genes. This process, called cold acclimation, triggers complex biochemical and physiological changes in plant cells, including alterations in membrane fluidity and lipid composition, the accumulation of compatible solutes and the regulation of gene expression [46–48]. De-acclimation is an important reverse regulatory process that enables overwintering plants to percept warm temperatures and resume growth in spring. Although rice is not an overwintering plant, the dynamic changes in the majority of chloroplast photosynthetic- and ribosome-associated proteins in DC90 and 9311 during the transition from chilling to recovery appeared to reflect the phenomena of cold acclimation and de-acclimation at the proteomic level (Fig. 6a, b; Table 2; Additional file 10). Intriguingly, the dynamic changes in these proteins during the recovery stage were distinct between the two genotypes. In 9311, both the ribosome- and photosynthesis-associated DEPs were incapable of regaining their normal abundance, indicating that seemed proteomic de-acclimation in 9311 was impaired severely even when the plants were restored to optimal conditions (Fig. 6a). However, strikingly, the majority of DC90 DEPs, in contrast to those of 9311, returned to normal abundance during recovery, indicating that the DC90 plants underwent proteomic cold acclimation and de-acclimation regulatory process during the whole period of the experimental regime (Fig. 6b). Therefore, these processes might facilitate the survival of rice plants under severe chilling stress.

### Imbalance of H_2_O_2_ production and scavenging may be responsible for impairing the de-acclimation of chloroplastic proteins in 9311

Oxidative burst, which is caused by the generation of large quantities of reactive oxygen species (ROS, e.g., O_2_^.-^, H_2_O_2_, OH˙, ^1^O_2_), is one of the earliest responses of plant cells to various environmental stresses [49]. ROS accumulation under environmental stresses may cause damage to lipids, DNA, and proteins; ROS also act as signaling molecule to trigger downstream molecular regulation processes, including ROS scavenging and DNA/protein damage repair. Therefore, the balance between ROS production and the activities of antioxidative enzymes determines whether oxidative signaling and/or damage will occur [50]. DAB and NBT staining showed ROS accumulation in both genotypes at an early timepoint in the chilling treatment (12 h) (Fig. 7a; Additional file 11 A, B). However, our study showed no significant differences in superoxide accumulation and scavenging between DC90 and 9311 at the tested timepoints (Fig. 7a, d), whereas the scavenging of H_2_O_2_ was distinctly different between DC90 and 9311. The accumulated H_2_O_2_ in DC90 was gradually scavenged beginning at the 36-h timepoint in the chilling treatment, whereas the increasing accumulation of H_2_O_2_ occurred from 12 to 72 h in 9311, resulting in cell death at the later timepoints (84–120 h). This result was confirmed by the analysis of the activity of H_2_O_2_ scavenging enzyme, catalase. The activity of CAT in 9311 was significantly down-regulated after 60 h of chilling treatment, whereas there was not significantly induced in DC90 (Fig. 7a, b). This in line with three ROS scavenging associated DEPs, prx11 (Q9FYP0), CATB (Q0D9C4), and catalase (Q10S82), which were specifically identified in 9311 in LC-MS/MS. They were all down-regulated under chilling stress. This behavior indicated that DC90 can maintain the balance between H_2_O_2_ production and elimination and survive severe chilling stress. The ability to scavenge ROS and reduce their damaging effects may be related to the regulatory mechanism of cold acclimation and de-acclimation at the proteomic level in wild rice.

### Cold acclimation and de-acclimation of photosynthetic and ribosomal proteins plays a vital role in the survival of rice under chilling stress

**Fig. 8:**
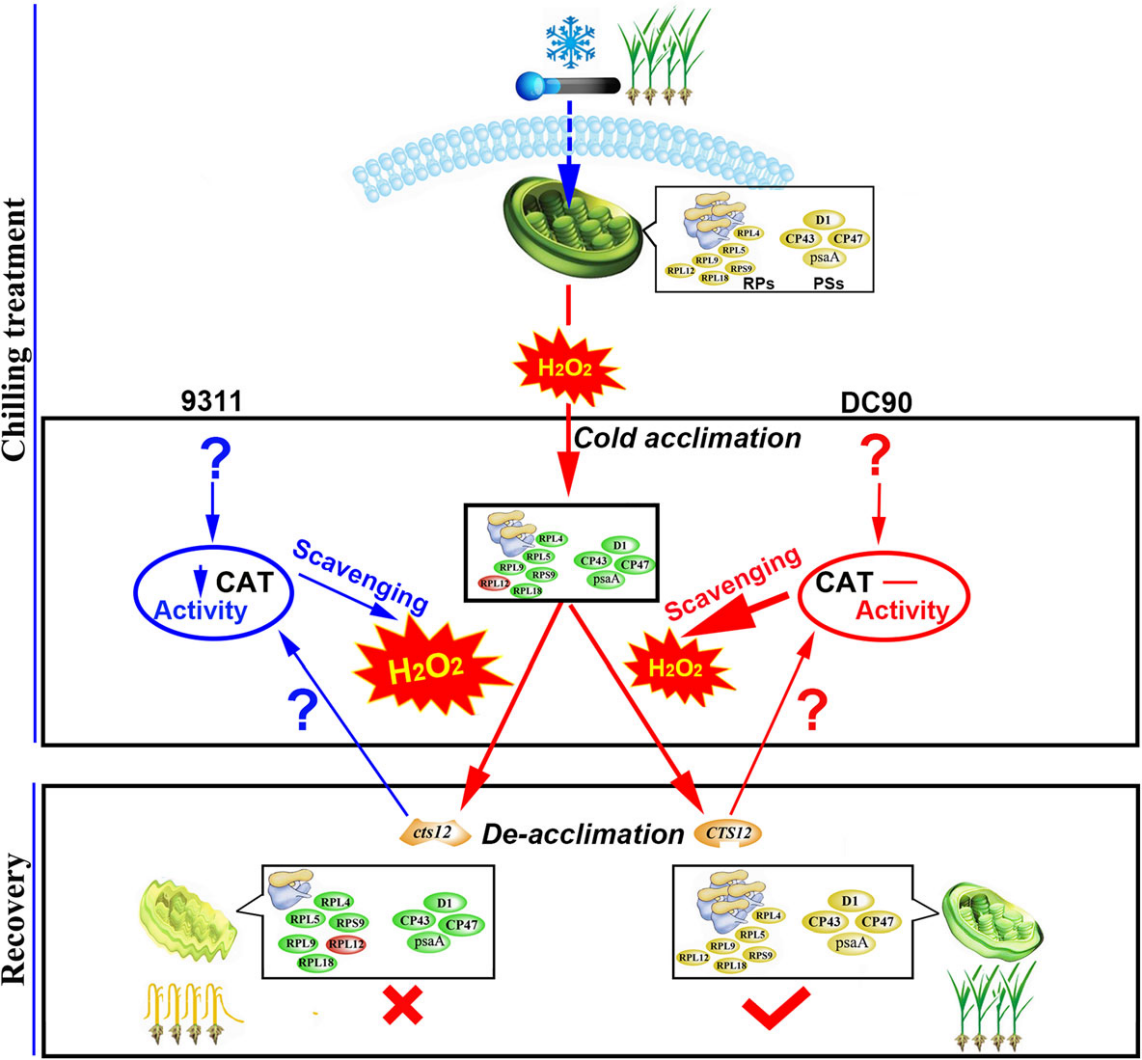
Working model proposed to depict the *CTS-12*-mediated mechanism underlying the chilling tolerance conferred by common wild rice. Yellow ellipses designate the abundance of proteins at the optimal temperature. Green ellipses designate the down-regulated abundance of proteins in the chilling or recovery stage. Red ellipses designate the up-regulated abundance of proteins in the chilling or recovery stage. RPs designates ribosomal proteins; PSPs designates photosystem proteins

In summary, a working model was proposed to depict the proteomic cold acclimation and de-acclimation in rice cells adapting to severe chilling stress and to speculate on the role of *CTS-12* in these processes (Fig. 8). When rice plants are exposed to low temperature, the cell membranes percept the chilling stress and trigger a decrease in the rate of protein translation and photosynthetic activity, which manifests as the down-regulation of chloroplast photosynthetic and ribosomal proteins. This down-regulation appeared to be an important process in the cold acclimation of rice plants. When the adverse environment is removed, the capability of de-acclimation of rice plants will survive chilling stress. The inhibition of photosynthetic activity reduces the utilization of absorbed light energy. The excess light energy results in the generation of toxic ROS, which in turn triggers photodamage to photosystem proteins, such as D1, a core component of photosystem II [51]. Damaged D1 is removed by FtsH proteolytic degradation [52–54]. Therefore, the turnover of D1 protein is thought to be a key regulatory step in the PSII repair cycle [55]. In this study, ribosomal proteins, which are constituents of the protein translation apparatus, were significantly inhibited by chilling stress, and thereby inhibited the turnover of D1 protein. That is, the balance between ROS scavenging and production must be maintained for the de-acclimation of rice plants when the adverse environment is removed. In 9311, the imbalance of H_2_O_2_ production and scavenging may cause the interruption of D1 protein turnover and cell death, and ultimately impair de-acclimation of rice plants when the adverse environmental factors are removed. Thus, we speculate that the major QTL, *CTS-12,* might directly or indirectly mediate the process of cold acclimation and de-acclimation of photosynthetic and ribosomal proteins in wild rice under severe chilling stress and recovery stage, respectively. Moreover, the differences of H_2_O_2_ production and scavenging between 9311 and DC90 arouse us to question what’s the possible correlation between H_2_O_2_ balance and *CTS-12-*mediated the cold acclimation and de-acclimation of photosynthetic and ribosomal proteins in rice under chilling stress. This will be addressed in our further investigation.

## Conclusion

Using iTRAQ-based proteomic analysis, we compared a chilling tolerance CSSL, DC90 with its chilling sensitive recurrent parent, 9311 under chilling stress and recovery treatment. In our results, 206 and 155 DEPs were identified in 9311 and DC90, respectively. Strikingly, the majority of which were enriched in the ‘structural constituent of ribosome’, ‘protein-chromophore linkage’, and ‘photosynthesis and light harvesting’ categories. And STEM analysis based on these DEPs revealed distinct dynamic responses of both chloroplast photosynthetic and ribosomal proteins between 9311 and DC90. Based on the proteomic analysis and physiological investigation of ROS in 9311 and DC90, we proposed that *CTS-12*, a QTL conferred DC90 chilling tolerance, might mediate the dynamic response of chloroplast photosynthetic and ribosomal proteins under chilling and recovery, and thereby enhancing the chilling stress tolerance of wild rice. Furthermore, the balance of H_2_O_2_ production and elimination in rice plants may play a certain role in maintaining the dynamic response of chloroplast photosynthetic and ribosomal proteins in whole period of chilling and recovery stress (cold acclimation / de-acclimation). The identified DEPs and the involvement of *CTS-12* in mediating the dynamic response of DC90 at the proteomic level illuminate and deepen the understanding of the mechanisms that underlie chilling stress tolerance in wild rice.

## Additional files

Additional file 1: Phenotype of 72-h chilling-treated/72-h recovery-treated seedlings of DC90 and 9311. A, The morphology of seedlings after 72-h recovery treatment; B, The percentage of the seedlings with yellowish leaf tips. Arrows in A indicate yellowish leaf tips. (TIF 9773 kb)

Additional file 2: Chilling-tolerant phenotypes of DC90 and 9311 under hydroponic culture conditions. A, DC90 and 9311 seedlings before chilling treatment; B, DC90 and 9311 seedlings after 5-day chilling and 7-day recovery treatment. Scale bar = 10 cm. (TIF 2700 kb)

Additional file 3: The list of proteins detected using iTRAQ labeling LC-MS/MS. (XLSX 2619 kb)

Additional file 4: Relative fold changes of differentially expressed proteins identified in this study. (XLSX 2862 kb)

Additional file 5: Summary of LC-MS/MS data. (TIF 2967 kb)

Additional file 6: Hierarchical clustering of three replicates of quantified proteins in chilling- and recovery-treated samples of DC90 and 9311. CSL1, CSL2, and CSL3 represent the 0-h, 60-h chilling-treated, and 60-h recovery-treated samples of 9311, and CTL1, CTL2, and CTL3 represent the 0-h, 60-h chilling-treated, and 60-h recovery-treated samples of DC90, respectively. C-60 h and R-60 h indicate the chilling- and recovery-treated stages, respectively. R1, R2, and R3 represent three replicates. (TIF 2694 kb)

Additional file 7: The primer sequences of corresponding DEPs-encoding genes and *GAPDH* using for qPCR. (XLSX 12 kb)

Additional file 8: GO enrichment analysis of DEPs identified in chilling stress and recovery treatment stages of DC90 and 9311 by comparison with its untreated control (*P* < 0.05). (XLSX 20 kb)

Additional file 9: Profile model analysis of all DEPs identified during the whole period of the chilling and recovery treatment of DC90 and 9311. The number at the bottom-left corner represents the number of DEPs assigned to the corresponding model. Colored profiles indicate a statistically significant number of genes assigned to that category. *P* < 0.05 was set as the significance level with Bonferroni correction. (TIF 6209 kb)

Additional file 10: The DEPs common to 9311 and DC90 in response to chilling stress and recovery treatment by comparison with untreated controls (XLSX 41 kb)

Additional file 11: ROS accumulation, the activity of the ROS scavenging related enzymes, and physiological changes in leaf tissues of DC90 and 9311 under chilling stress and recovery conditions. A, DAB staining of DC90 and 9311 leaf samples under recovery condition; B, NBT staining of DC90 and 9311 leaf samples under recovery condition; C, The relative electrolyte leakage in leaf tissues of DC90 and 9311 during chilling stress and recovery conditions; D, The MDA content in leaf tissues of DC90 and 9311 during chilling stress and recovery conditions; E, The APX activity in leaf tissues of DC90 and 9311 during chilling stress and recovery conditions. Scale bar = 4 mm in A, B. Data in C, D, and E are shown as means ± SD (*n* = 3). Different letters at top of each column indicate a significant difference at *P* < 0.05 determined by Tukey’s HSD test. (TIF 9074 kb)

## Abbreviations

APX: Ascorbate peroxidase
AsA: Ascobic acid
BP: Biological process
CAT: Catalase
CC: Cellular component
CSL: Chilling-sensitive line
CTL: Chilling-tolerant line
DAB: 3,3′-Diaminobenzidine
DEPs: Differentially expressed proteins
FDR: False discovery rate
IAM: Iodoacetamide
iTRAQ: Isobaric tags for relative and absolute quantification
MDA: Malonaldehyde
MF: Molecular function
NBT: Nitroblue tetrazolium
POD: Peroxidase
RFC: Relative fold change
SOD: Superoxide Dismutase
STEM: Short Time-series Expression Miner
TEAB: Tetraethylammonium bromide
