# Supplementary figures and images for: Comparative proteomic analysis of QTL CTS-12 derived from wild rice (Oryza rufipogon Griff.), in the regulation of cold acclimation and de-acclimation of rice (Oryza sativa L.) in response to severe chilling stress
Source: BMC Plant Biol. 2018 Aug 10;18:163. doi: 10.1186/s12870-018-1381-7 (PMC6086036; doi:10.1186/s12870-018-1381-7)

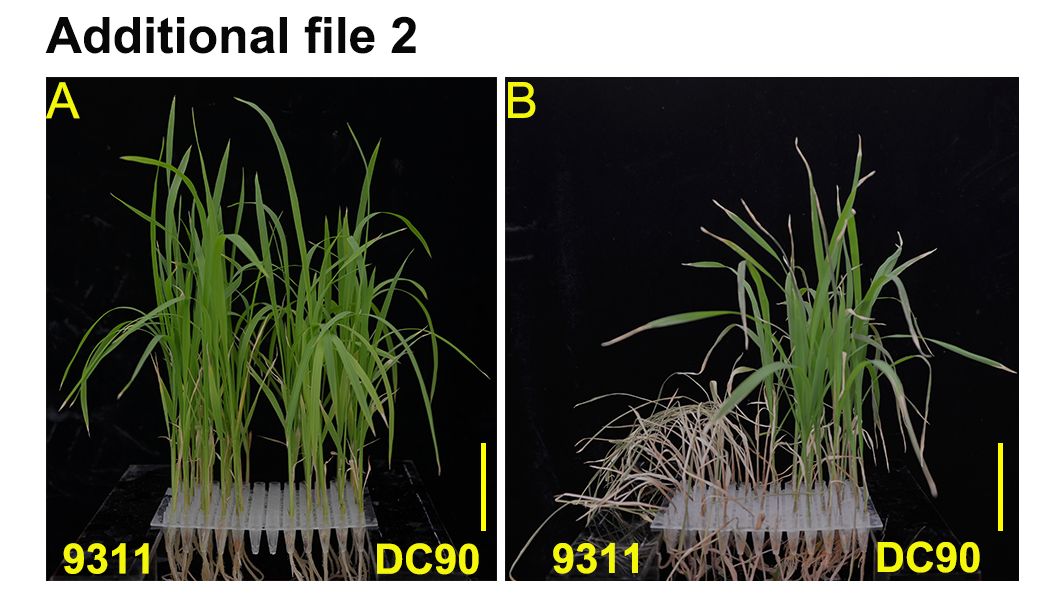

Supplement: Supplementary file 2 — Chilling-tolerant phenotypes of DC90 and 9311 under hydroponic culture conditions. A, DC90 and 9311 seedlings before chilling treatment; B, DC90 and 9311 seedlings after 5-day chilling and 7-day recovery treatment. Scale bar = 10 cm. (TIF 2700 kb) [file 12870_2018_1381_MOESM2_ESM.tif]

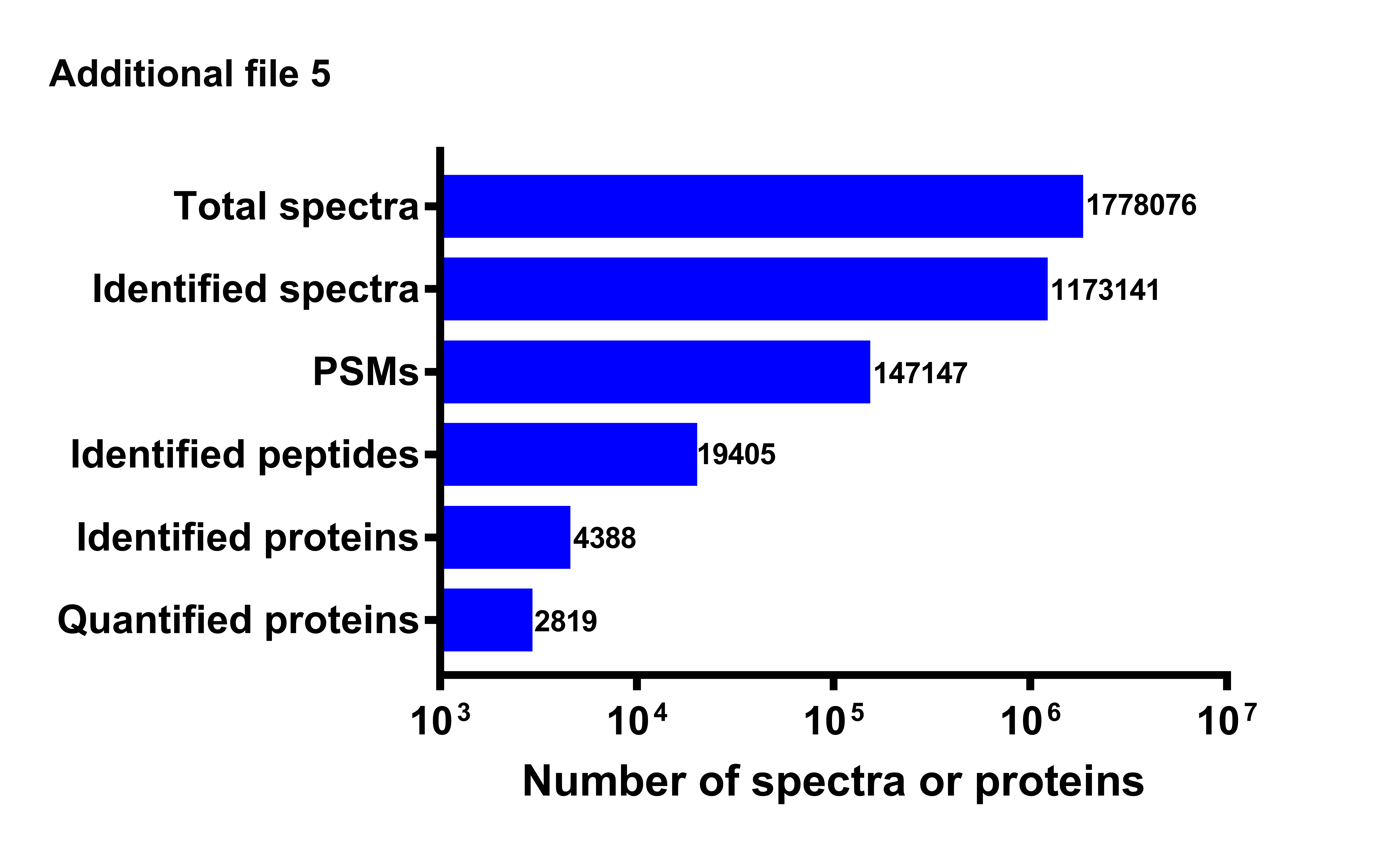

Supplement: Supplementary file 5 — Summary of LC-MS/MS data. (TIF 2967 kb) [file 12870_2018_1381_MOESM5_ESM.tif]

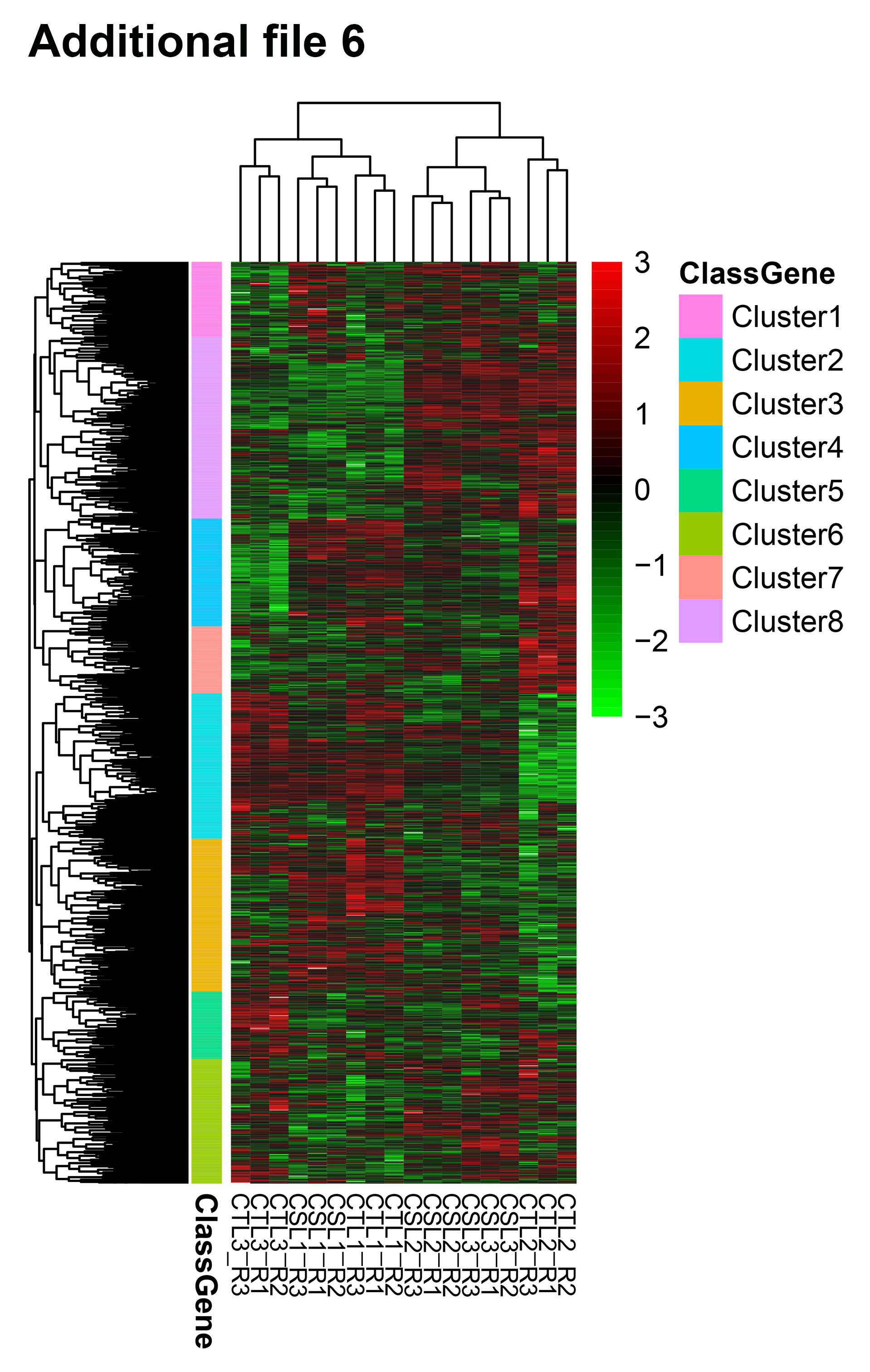

Supplement: Supplementary file 6 — Hierarchical clustering of three replicates of quantified proteins in chilling- and recovery-treated samples of DC90 and 9311. CSL1, CSL2, and CSL3 represent the 0-h, 60-h chilling-treated, and 60-h recovery-treated samples of 9311, and CTL1, CTL2, and CTL3 represent the 0-h, 60-h chilling-treated, and 60-h recovery-treated samples of DC90, respectively. C-60 h and R-60 h indicate the chilling- and recovery-treated stages, respectively. R1, R2, and R3 represent three replicates. (TIF 2694 kb) [file 12870_2018_1381_MOESM6_ESM.tif]

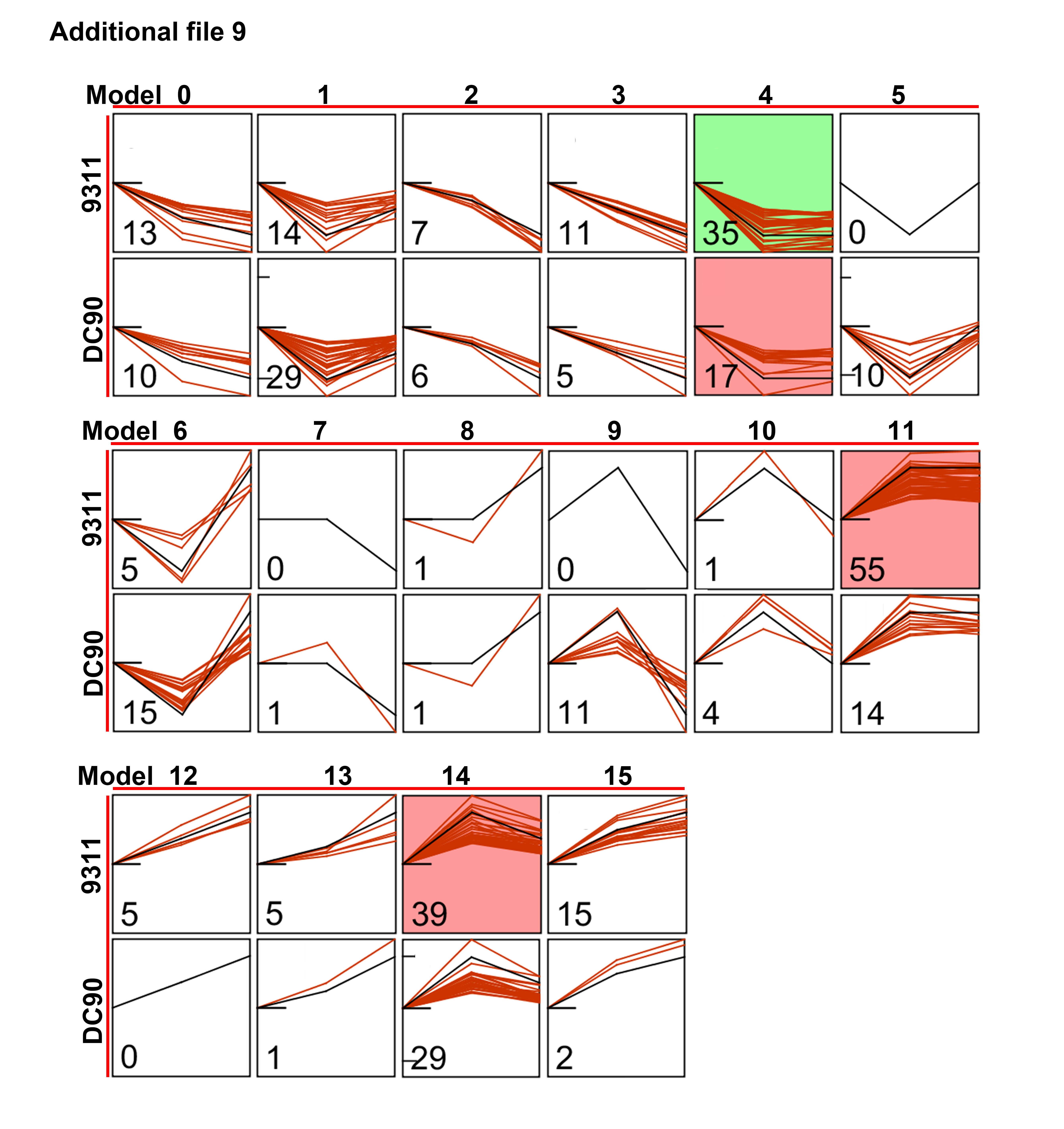

Supplement: Supplementary file 9 — Profile model analysis of all DEPs identified during the whole period of the chilling and recovery treatment of DC90 and 9311. The number at the bottom-left corner represents the number of DEPs assigned to the corresponding model. Colored profiles indicate a statistically significant number of genes assigned to that category. P < 0.05 was set as the significance level with Bonferroni correction. (TIF 6209 kb) [file 12870_2018_1381_MOESM9_ESM.tif]
